# Third-generation anti-CD19 CAR T cells for relapsed/refractory chronic lymphocytic leukemia: a phase 1/2 study

**DOI:** 10.1038/s41375-024-02392-7

**Published:** 2024-08-27

**Authors:** Patrick Derigs, Maria-Luisa Schubert, Peter Dreger, Anita Schmitt, Schayan Yousefian, Simon Haas, Caroline Röthemeier, Brigitte Neuber, Angela Hückelhoven-Krauss, Monika Brüggemann, Helga Bernhard, Guido Kobbe, Albrecht Lindemann, Mathias Rummel, Birgit Michels, Felix Korell, Anthony D. Ho, Carsten Müller-Tidow, Michael Schmitt

**Affiliations:** 1 Internal Medicine V, Hematology, Oncology and Rheumatology, Heidelberg University Hospital, Heidelberg, Germany; 2 Berlin Institute of Health (BIH) at Charité – Universitätsmedizin Berlin, Berlin, Germany; 3 Berlin Institute for Medical Systems Biology, Max Delbrück Center for Molecular Medicine in the Helmholtz Association, Berlin, Germany; 4 Department of Hematology, Oncology and Tumor Immunology, Charité University Medicine, Berlin, Germany; 5 German Cancer Consortium (DKTK) and German Cancer Research Center (DKFZ)/National Center for Tumor Diseases (NCT), Heidelberg, Germany; 6 Heidelberg Institute for Stem Cell Technology and Experimental Medicine (HI-STEM gGmbH), Heidelberg, Germany; 7 Precision Healthcare University Research Institute, Queen Mary University of London, London, UK; 8 Department of Internal Medicine II, University Hospital Schleswig-Holstein, Kiel, Germany; 9 Department of Internal Medicine V, Klinikum Darmstadt, Darmstadt, Germany; 10 Department of Hematology, Oncology and Clinical Immunology, University Hospital Düsseldorf, Düsseldorf, Germany; 11 Onkologie in Ettlingen, Ettlingen, Germany; 12 Department of Internal Medicine IV, University Hospital Giessen, Giessen, Germany

**Keywords:** Phase I trials, Phase II trials, Chronic lymphocytic leukaemia, Immunotherapy, Translational research

## Abstract

Third-generation chimeric antigen receptor T cells (CARTs) for relapsed or refractory (r/r) chronic lymphocytic leukemia (CLL) may improve efficacy compared to second-generation CARTs due to their enhanced CAR design. We performed the first phase 1/2 investigator-initiated trial evaluating escalating doses of third-generation CARTs (HD-CAR-1) targeting CD19 in patients with r/r CLL and B-cell lymphoma. CLL eligibility criteria were failure to two therapy lines including at least one pathway inhibitor and/or allogeneic hematopoietic cell transplantation. Nine heavily pretreated patients received HD-CAR-1 at dose levels ranging from 1 × 10^6^ to 200 × 10^6^ CART/m^2^. In-house HD-CAR-1 manufacturing was successful for all patients. While neurotoxicity was absent, one case of grade 3 cytokine release syndrome was observed. By day 90, six patients (67%) attained a CR, five of these (83%) with undetectable MRD. With a median follow-up of 27 months, 2-year PFS and OS were 30% and 69%, respectively. HD-CAR-1 products of responders contained significantly more CD4 + T cells compared to non-responders. In non-responders, a strong enrichment of effector memory-like CD8 + T cells with high expression of CD39 and/or CD197 was observed. HD-CAR-1 demonstrated encouraging efficacy and exceptionally low treatment-specific toxicity, presenting new treatment options for patients with r/r CLL. Trial registration: #NCT03676504.

## Introduction

The use of second-generation CD19-directed chimeric antigen receptor T-cell (CART) therapy has revolutionized the treatment of several B-cell malignancies [1–9]. However, although chronic lymphocytic leukemia (CLL) has been the disease used for proof of principle of CART efficacy in humans [10], the clinical development of CLL CARTs has been hampered by inferior response rates and shorter response duration compared to other indolent B-cell lymphomas (ZUMA5, ELARA) [7, 11–13]. It has been hypothesized that this is at least in part due to the inherent dysfunction and alterations of T cells in this disease [10, 14–19]. However, liso-cel has recently been approved by the U.S. Food and Drug Administration as the first second-generation CART product for adults with relapsed or refractory (r/r) CLL or small lymphocytic lymphoma.

CARTs derived from patients with CLL typically display signs of exhaustion that lead to restricted expansion and decreased cytokine production resulting in insufficient leukemia suppression [20]. One way of improving CART efficacy in CLL involves modifications of the CAR vector. Third-generation CARs harbor two costimulatory domains mediating enhanced and faster expansion as well as longer persistence of CARTs [21–25]. However, clinical data on third-generation CARTs for CLL are scarce [26].

Here, we report initial findings of third-generation CARTs manufactured academically, as part of the investigator-initiated trial (IIT) Heidelberg CAR T-cell trial 1 (HD-CAR-1) [27], for the treatment of r/r CLL.

## Methods

### Trial design and manufacturing of CARTs

This was a 2-strata basket trial in patients with r/r acute lymphoblastic leukemia (ALL, stratum 1), and B-cell lymphoma and CLL (stratum 2) aiming to assess feasibility and to identify dose-limiting toxicities of HD-CAR-1 CARTs. To be eligible, patients with CLL needed to have failed ≥2 lines of therapy, including at least one pathway inhibitor and/or allogeneic hematopoietic cell transplantation (alloHCT), and an Eastern Cooperative Oncology Group performance score of 0 or 1. In a 3 + 3 design (across all eligible entities), patients received increasing doses of autologous T cells retrovirally transduced with a third-generation CD19-directed CAR (RV-SFG.CD19.CD28.4-1BBzeta) [27]. HD‐CAR‐1 CARTs were manufactured as previously described [27–29] at the institutional Good Manufacturing Practice Core Facility. For transduction, RV-SFG.CD19.CD28.4-1BBzeta retroviral vector supernatant was provided by Prof. Malcolm Brenner from Baylor College of Medicine in Houston, Texas, USA. This CAR harbors the costimulatory domains CD28 and 4-1BB. The trial was approved by the institutional review board as well as by the German federal regulatory authority for immunotherapy (Paul-Ehrlich-Institut, Langen, Germany), and written informed consent was obtained from all participants. The trial was conducted in compliance with the principles of the Declaration of Helsinki.

### CART therapy and endpoint evaluation

Patients received HD-CAR-1 CARTs 2 days following administration of lymphodepletion with fludarabine 30 mg/m^2^/d and cyclophosphamide 500 mg/m^2^/d for 3 days. Cytokine release syndrome (CRS), immune effector cell-associated neurotoxicity syndrome (ICANS), and immune effector cell-associated hematotoxicity (ICAHT) were graded according to consensus guidelines [30, 31]. CRS and ICANS were managed according to institutional guidelines as published [32]. Adverse events were graded according to the National Cancer Institute Common Terminology Criteria for Adverse Events, version 5.0. For lymphodepletion, CART infusion and monitoring patients were hospitalized from day −6 through day +14. The clinical efficacy of HD-CAR-1 treatment was assessed using the 2018 International Workshop on Chronic Lymphocytic Leukemia (iwCLL2018) criteria [33]. Minimal or measurable residual disease (MRD) was assessed by MRD-flow using one CLL cell in 10.000 events as detection threshold [34]. HD-CAR-1 CART frequencies were assessed as described [35].

### Analysis of cellular composition of CART products with flow cytometry

HD-CAR-1 CART products from seven CLL patients (#1–7) were analyzed with spectral flow cytometry using a 36-marker panel on two different days (day 1: samples of #1-5 and #7, day 2: sample of #6). In brief, samples were thawed, washed and stained with the antibody mix. Staining was conducted over three consecutive rounds, with each round consisting of a 20-min incubation at 4 °C (antibodies used summarized in Supplementary Table S1). Samples were measured on Cytek Aurora flow cytometer (Cytek Biosciences, Fremont, CA, USA).

### Computational analysis

Spectral unmixing was performed using SpectroFlo (Cytek Biosciences). To detect and remove anomalies based on common flow cytometry parameters, .fcs files were further processed using the R package flowAI (version 1.24.0). The function flow_auto_qc was run, and anomalies identified based on the flow rate were removed. These .fcs files were then imported into FlowJo (version 10.8.1; BD Biosciences, Franklin Lakes, NJ, USA) to assess unmixing quality and for gating on single viable cells. The data was then transformed using the logicle transform function of FlowJo (export channel values) and exported as .csv files for downstream analysis in R (version 4.3.0). Since batch effects between the samples of #1-5 and #7 and the sample of #6 were observed, downstream analyses involving clustering and Uniform Manifold Approximation and Projection (UMAP) [36] visualization were performed for samples of #1-5 and #7 only. Cell type labels of the sample of #6 were determined by linear discriminant analysis (LDA) from the MASS package (version 7.3–60) [37] using cell type annotations of the samples of #1-5 and #7 as reference.

All downstream analyses were performed using the R package Seurat (version 5.0.3) [38]. In brief, this involved atomic sketching (a non-uniform downsampling approach), a dimensional reduction using principal component analysis (PCA) followed by Louvain clustering and visualization of the data in a UMAP embedding. Cluster labels of cells not included in the initial sketching were determined by LDA using the R package MASS. Clusters were manually annotated based on surface marker expression and expert knowledge. Cell type frequencies were calculated per sample and compared between non-responders (#1, #2 and #7) and responders (#3-6). In accordance with the iwCLL2018 criteria [33], responders were defined as patients who achieved a CR after CART infusion that was sustained at least for 2 months. All non-responders had undergone prior alloHCT, while none of the responders had. Significance levels were determined by a two-sided Welch’s *t*-test. After overall cluster annotation, CD4+ and CD8 + T cells were separated and clustered independently using the same approach as described above. Differential abundance of cells within the respective UMAP space was highlighted by density plots. For quantification of differentially abundant CD4+ or CD8 + T-cell subtypes between responders and non-responders, the frequency of each T-cell subtype was calculated per sample. For each subtype, the frequency of responders was divided by the corresponding mean frequency of non-responders. These responder-specific fold-changes were log2 transformed and visualized in boxplots.

### Statistical analysis

Statistics were calculated using Prism Software (version 10.2.0; Graphpad Software Inc., Boston, MA, USA). Progression-free survival (PFS) was determined measuring the duration from the date of CART administration until clinical progression, relapse, or death. Survival curves were compared using log-rank testing. A *p*-value less than 0.05 was considered statistically significant.

## Results

### Patients

Eight patients with r/r CLL were enrolled between October 2018 and May 2023. As the trial cohort was already fully recruited, another patient with r/r CLL was treated compliant with the trial but formally off-study in July 2023 after having given fully informed consent (#9). Baseline characteristics of all nine patients are listed in Table 1. Patients had a median age of 60 years (range 45 to 68) and had received a median of 5 (range 2 to 10) prior treatment lines. Four patients (44%) had received prior alloHCT. Seven patients (78%) harbored TP53 abnormalities. All patients had failed Bruton’s tyrosine kinase inhibitors (BTKi), and all had received at least one venetoclax-based regimen. Eight of the nine patients were refractory to venetoclax-based treatment as well. Nevertheless, bridging therapy was administered to all patients, mostly with venetoclax-antibody combinations, resulting in CR and partial remission (PR) in three and two patients, respectively. However, all patients had flow-detectable MRD at lymphodepletion. One patient (#8) had a history of Richter transformation which however was not present at the time of enrollment.

**Table 1:** Baseline characteristics of all registered patients.

Patient #	Age (years)	Gender	Diagnosis to CART (years)	# Prior therapy lines	Prior therapy lines	Prior alloHCT/donor/source	TP53 abnormal	Bridging therapy	Lymph node involvement at LD	Disease status at LD
1	54	M	12.5	10	FC, BR, alemtuzumab, DHAP, alloHCT, Len, ofatumumab, Ibr, Ide, Ven	Yes/MRD*/PBSCT	+	Ven	Yes	PD MRD 0.5%
2	60	M	12.3	6	FCR, BR, alemtuzumab, alloHCT, Ibr, VenR	Yes/MUD/PBSCT	+	VenR	Yes	SD MRD 16%
3	62	M	10.5	5	BR, Ibr, Ven, IdeR, GVen	No	+	Ven	No	CR MRD 3.8%
4	64	M	7.1	4	BR, Ibr, VenR, G	No	−	Ibr	No	CR MRD 0.01%
5	68	M	12.8	5	FCR, BR, Ibr, VenR, GVen	No	+	GVen	Yes	PR MRD 21%
6	63	F	4.0	2	Ibr, VenR	No	+	Ven	No	CR MRD 0.4%
7	56	F	19.7	10	FC, FCM, BR, R-HAM, alloHCT, ofatumumab, Len, Ibr, Ven, GVen	Yes/MUD/PBSCT	+	GVen	No	PR
8	45	F	6.2	6	R-CHOP, R-DHAP, autoHCT, IbrR, VenR, alloHCT, IbrVen	Yes/MUD/PBSCT	+	IbrVen	No	PD
9	54	M	6.3	4	GIVe, GAVe, VenR	No	−	VenR	Yes	PD

*alloHCT* allogeneic hematopoietic cell transplantation, *autoHCT* autologous hematopoietic cell transplantation, *B* bendamustine, *CR* complete remission, *DHAP* dexamethasone + high-dose cytarabine + cisplatin, *F* female, *FC* fludarabine + cyclophosphamide, *FCM* fludarabine + cyclophosphamide + mitoxantrone, *FCR* fludarabine + cyclophosphamide + rituximab, *G* obinutuzumab, *GAVe* obinutuzumab + acalabrutinib + venetoclax, *GIVe* obinutuzumab + ibrutinib + venetoclax, *Ibr* ibrutinib, *Ide* idelalisib, *LD* lymphodepletion, *Len* lenalidomide, *M* male, MRD* HLA-matched related donor, *MRD* minimal/measurable residual disease, *MUD* HLA-matched unrelated donor, *PBSCT* peripheral blood stem cell transplantation, *PD* progressive disease, *PR* partial remission, *R* rituximab, *R-CHOP* rituximab + cyclophosphamide + doxorubicin + vincristine + prednisone, *R-DHAP* rituximab + dexamethasone + high-dose cytarabine + cisplatin, *R-HAM* rituximab + high-dose cytarabine + mitoxantrone, *SD* stable disease, *Ven* venetoclax.

### Manufacturing and dosing of HD-CAR-1 CARTs

HD-CAR-1 manufacturing was successful in all patients with a median transduction efficiency of 52.3% (range 41.7% to 67.7%) and a median CART viability of 95.2% (range 86.2% to 95.8%). Median duration of manufacturing was 13 days (range 10 to 13). A second leukapheresis had to be performed in two patients (#3 and #5) due to an unfavorable T-cell:B-cell ratio in the first leukapheresis products. Characteristics and cellular composition of HD-CAR-1 CART products are listed in Supplementary Table S2.

All patients received at least one dose of HD-CAR-1 CARTs (Table 2). Dose levels (DL) were DL1 (1 × 10^6^ CARTs/m^2^) in one patient, DL2 (5 × 10^6^ CARTs/m^2^) in one patient, DL5 (10 × 10^7^ CARTs/m^2^) in two patients and DL6 (20 × 10^7^ CARTs/m^2^) in five patients.

**Table 2:** Toxicity and outcome of all patients treated with HD‐CAR‐1 CARTs.

Patient #	Dose level [CARTs/m^2^]	CRS grade	Toci doses	Dexa days	ICANS grade	Early ICAHT grade	Infections after CART until last f/u [day after CART]	CART peak (copies/µg PBMC DNA)	Best response [day after CART]	Disease status at LD → Best response [MRD change]
1	1 [1 × 10^6^]	1	0	0	0	2	respiratory^a^ [day 238, 464], *E. coli* sepsis [day 902]	0	SD MRD 0.03% (PB) [day 90]	PD MRD 0.5% → SD MRD 0.03% [-94.0%]
2	2 [5 × 10^6^]	3	3	6	0	2	respiratory^a^ [day 122]	37,792	SD MRD 0.04% (PB) [day 90]	SD MRD 16% → SD MRD 0.04% [-99.8%]
3	5 [10 × 10^7^]	2	1	0	0	2	COVID-19 [day 426]	369,756	CR uMRD (PB) [day 91]	CR MRD 3.8% → CR uMRD [-100%]
4	5 [10 × 10^7^]	0	0	0	0	3	–	46,071	CR uMRD (PB + BM) [day 91]	CR MRD 0.01% → CR uMRD [-100%]
5	6 [20 × 10^7^]	2	3	8	0	4	respiratory^a^ [day 5], HSV-1 (lower lip) [day 21]	179,079	CR MRD 0.02% [day 85]	PR MRD 21% → CR MRD 0.02% [-99.9%]
6	6 [20 × 10^7^]	0	0	0	0	1	–	52,965	CR uMRD (PB + BM) [day 90]	CR MRD 0.4% → CR uMRD [-100%]
7	6 [20 × 10^7^]	2	1	0	0	2	CMV reactivation [day 618], COVID-19 [day 740], *C. diff*. colitis [day 798], respiratory^a^ [day 800]	219,586	CRu uMRD (PB) [day 90]	PR → CRu uMRD [-100%]
8	6 [20 × 10^7^]	1	0	0	0	1	–	82,358	CR uMRD (PB + BM) [day 90]	PD → CR uMRD [-100%]
9	6 [20 × 10^7^]	1	0	0	0	2	–	198,376	SD MRD 2.5% [day 43]	PD → SD MRD 2.5%

^a^no pathogen identified.

*BM* bone marrow, *CMV* cytomegalovirus, *COVID-19* coronavirus disease 2019, *CR* complete remission, *CRS* cytokine release syndrome, *CRu* CR unconfirmed, *Dexa* dexamethasone, *f/u* follow-up; *HSV-1* herpes simplex virus type 1, *ICAHT* immune effector cell-associated hematotoxicity, *ICANS* immune effector cell-associated neurotoxicity syndrome, *LD* lymphodepletion, *MRD* minimal/measurable residual disease, *MUD* matched unrelated donor, *PB* peripheral blood, *PD* progressive disease, *PR* partial remission, *SD* stable disease, *Toci* tocilizumab, *uMRD* undetectable MRD.

### Safety

HD-CAR-1 CARTs were well tolerated (Table 2). Although seven of nine patients experienced CRS, higher grade CRS was observed only in a single patient (11%), and ICANS was completely absent. Early ICAHT occurred in eight patients (89%), but was grade 4 only in a single patient. Late ICAHT was observed as grade 1 in one patient (#9) and as grade 2 in two patients (#2 and #7) without the need for granulocyte colony-stimulating factor support.

**Fig. 1:**
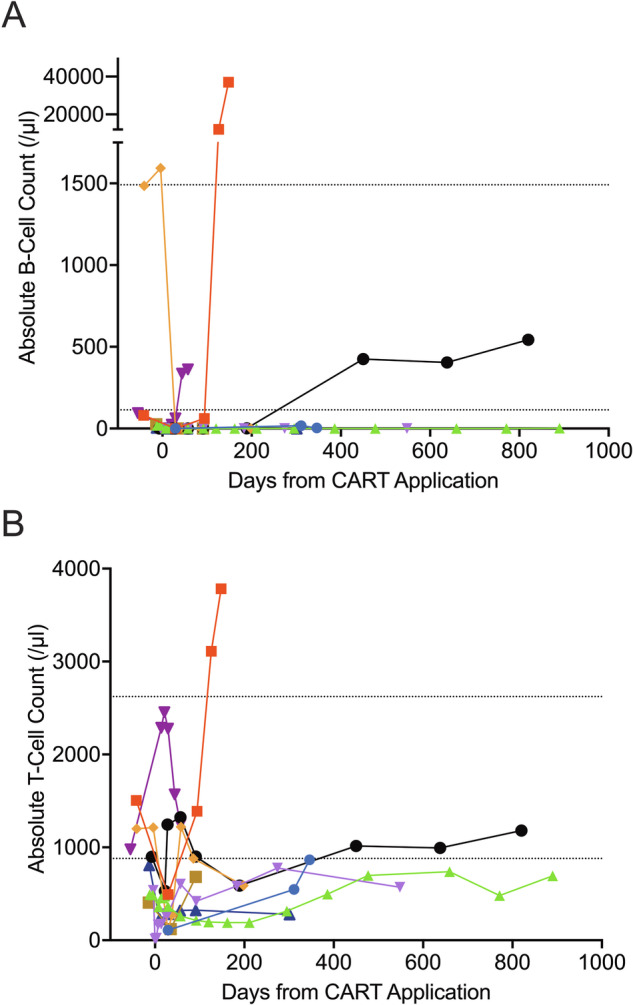
Absolute B-cell and T-cell counts in the peripheral blood (PB). **A** Absolute B-cell count in cells/μl. **B** Absolute T-cell count in cells/μl. PB of patients was assessed with flow cytometry directly before and up to 890 days after CART administration. Normal ranges of absolute values are displayed according to [39].

All six patients with CR as best response presented B-cell aplasia at end-of-study (EOS) on day 90 after CART administration (#3-8; Fig. 1A). Two patients showed absolute T-cell counts above the lower limit of normal (LLN) according to [39] at EOS, but presented counts below LLN at day 200 after CART administration (#5 and #6; Fig. 1B). In one patient, B-cell and T-cell recovery took place on day 450 besides ongoing CR with undetectable MRD (uMRD; #6). B-cell aplasia and lymphopenia were still present in patients #3, #4, #5, and #9 at their latest assessment on day 771, 548, 198 and 301 after CART administration, respectively (Fig. 1).

Hypogammaglobulinemia was observed in eight of nine patients at lymphodepletion. One of these patients showed recovery of gamma globulin levels after HD-CAR-1 treatment (#5). One patient (#2) did not exhibit hypogammaglobulinemia at lymphodepletion or after day 90 post CART infusion. Intravenous immunoglobulins were administered to three patients following HD-CAR-1 treatment (#1, #3 and #5).

The only early infections post-CART infusion occurred in patient #5 who presented herpes simplex virus type 1 infection of the lower lip and respiratory infection without pathogen identification. Late infections (beyond day 30) occurred in four patients (#1-3 and #7; Table 2).

### Expansion and persistence of HD-CAR-1

**Fig. 2:**
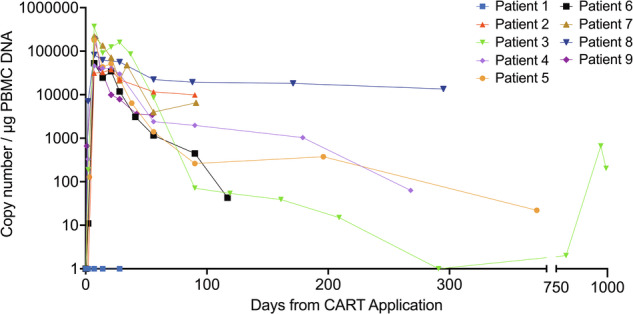
Expansion of HD-CAR-1 CARTs in the peripheral blood (PB). Rapid expansion of CARTs was observed in eight of nine patients. CART expansion in PB was measured by single‐copy gene duplex quantitative PCR (SCG‐DP‐PCR) as described [35].

Rapid CART expansion in the peripheral blood (PB) was observed in eight patients (89%) with a median CART peak level of 82,358 CART/µg peripheral blood mononuclear cell (PBMC) DNA (range 37,792 to 369,756; Table 2). In all seven patients evaluated, CARTs were persistent at EOS. Five patients were evaluated beyond 100 days after HD-CAR-1 infusion, with detectable CARTs in the PB of all patients (#3-6 and #8; Fig. 2). Patient #3 still showed 202 CART/µg PBMC DNA even at day 994 after HD-CAR-1 infusion, and in patient #8 a particularly high CART concentration was observed with 18,199 and 13,521 CART/µg PBMC DNA at day 171 and 295, respectively.

### Outcomes

All patients reached EOS on day 90 after CART administration (Table 2). Whereas clinically meaningful responses were not observed at dose levels 1 and 2, CR as best response was achieved with higher CART dose levels in six of seven patients (86%), with uMRD in five of them (72%). In #7, CR could not be confirmed. Although it has to be noted that five patients already had responded to bridging therapies at lymphodepletion, all of these deepened response after HD-CAR-1 treatment, either from CR MRD+ to CR uMRD, or from PR to CR MRD + /uMRD (Table 2). Median duration of response (DOR) was 6.4 months.

**Fig. 3:**
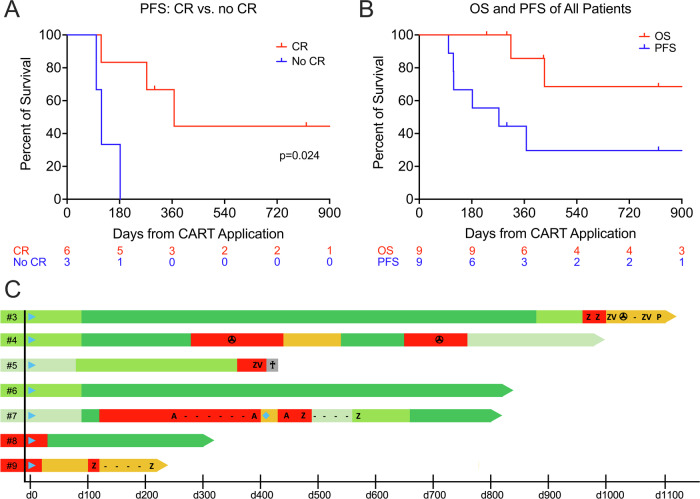
Outcome of patients after treatment with HD-CAR-1. **A** Progression-free survival (PFS) of all patients achieving a complete remission (CR) after CART administration vs. non-CR patients. **B** Overall survival (OS) and PFS of all patients after CART treatment. **C** Swimmer plots of all patients after CART administration treated at dose level 5 or higher. : CART therapy; : progressive disease; : stable disease; : partial remission; : MRD-positive CR; : MRD-negative CR; : death; : 2nd allogeneic hematopoietic cell transplantation; : radiation therapy; A acalabrutinib, P pirtobrutinib, V venetoclax, Z zanubrutinib.

PFS was significantly longer in patients who achieved a CR versus those who did not (median PFS, 12.1 months vs. 3.8 months, p 0.024; Fig. 3A). With a median follow-up of 27 months, 2-year PFS and OS for all patients were 30% and 69%, respectively (Fig. 3B). Course of the disease after CART administration of all patients treated at dose level 5 or higher is presented in Fig. 3C. All cases of CLL relapse or progression after HD-CAR-1 treatment were found to be CD19-positive. Three patients died due to PD, four patients are alive after disease progression, and two patients are in ongoing MRD-negative CR (#6 and #8).

### Cellular composition of CART products of responders vs. non-responders

**Fig. 4:**
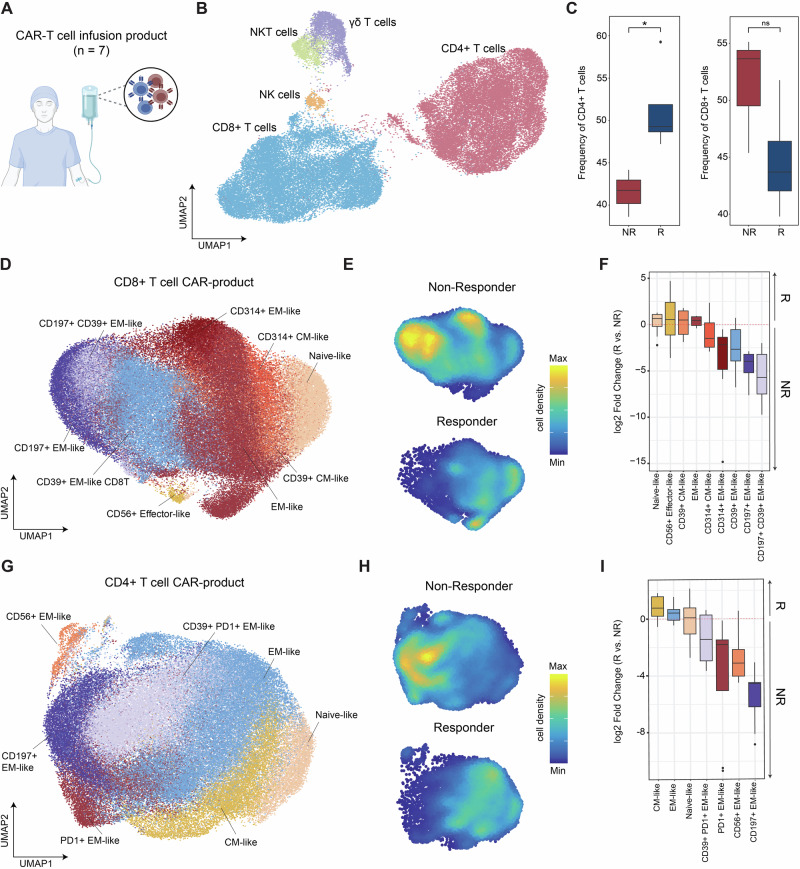
Cellular composition of HD‐CAR‐1 CART products of responders (*n* = 4) vs. non-responders (*n* = 3). **A** HD-CAR-1 CART products of seven CLL patients (#1-7) were analyzed with spectral flow cytometry using a 36-marker panel and computational analysis (see methods). **B** The downsampled subset of cells from all seven CART products is presented with uniform manifold approximation and projection (UMAP) visualization. Clusters are labeled depending on surface marker expression and displayed in different colors. **C** Frequencies of CD4+ (left) and CD8+ (right) T cells within the CART products of responders (R) vs. non-responders (NR) are displayed as bloxplots. Significance levels were determined by a two-sided Welch’s *t*-test. CD8+ **D** and CD4+ **G** T-cell subsets extracted from all seven CART products are presented with UMAP visualization and clusters are annotated based on surface marker expression and displayed in different colors. Differential compositions between R and NR of CD8+ **E** and CD4+ **H** T-cell subsets are displayed as density plots. Differential frequencies between R and NR of specific CD8+ **F** and CD4+ **I** T-cell subtypes are presented as boxplots of log2 fold-changes. Higher abundance in R is indicated as positive log2 fold change and negative log2 fold changes visualize higher frequencies in NR. CM central memory, EM effector memory, NK natural killer, NKT natural killer T cells, ns not significant.

To deeply characterize HD-CAR-1 CART products, we performed 36-plex spectral flow cytometry of CART products from four responders (#3-6) and three non-responders (#1, #2 and #7; Fig. 4A). Notably, all non-responders had previously received alloHCT, unlike the responders. As expected, CART products comprised mostly CD4+ and CD8 + T cells, albeit minor fractions of natural killer cells (NK), natural killer T cells (NKT) and γδ T cells were also found (Fig. 4B). Quantification of cell types revealed a significant enrichment of CD4 + T cells in responders compared to non-responders. Correspondingly, CD8 + T cells were more abundant in non-responding patients (Fig. 4C). Unsupervised clustering and dimensionality reduction of the CD8+ and CD4 + T-cell compartments (Fig. 4D, G) revealed additional differences in the composition of CART products between responders and non-responders as indicated by the shifts in cellular density within the respective T-cell compartment (Fig. 4E, H). Quantification of cell type abundances indicated a strong enrichment of effector memory (EM)-like CD8 + T cells with high expression of CD39 and/or CD197 in non-responders compared to responders (Fig. 4F). Similarly, non-responders displayed a higher fraction of EM-like cells in the CD4 + T-cell compartment with expression of exhaustion markers including CD39 and programmed cell death protein 1 (PD1) (Fig. 4I).

## Discussion

Over the past two decades, the landscape of CLL therapy has undergone a significant transformation, moving away from chemoimmunotherapy approaches towards the adoption of targeted therapies, notably BTK and B-cell lymphoma 2 inhibitors [10, 40]. However, challenges persist as in particular patients with genetically high-risk CLL tend to develop resistance to these targeted therapies with a consecutive dismal outlook [41–43]. It has been advised to consider these patients for alloHCT or exploratory cellular therapies [10, 40, 44].

Although CD19-directed CARTs have been studied in r/r CLL by various groups using various second-generation CART constructs [45–50], only few entered advanced phases of clinical development. This was largely due to comparably low rates of complete responses and durable disease control. Of the CD19 CARTs currently labeled for clinical use in other B-malignancies, only brexu-cel and liso-cel have been explored in CLL. Brexu-cel was administered to 15 heavily pretreated patients in the phase 1 ZUMA-8 clinical trial and resulted in an ORR of 47% (CR 13%) [51]. Liso-cel, a CART product that features a consistent 1:1 CD4 + :CD8 + CART ratio, was evaluated in the larger phase 1/2 TRANSCEND CLL 004 trial, which was the basis for the recent U.S. Food and Drug Administration approval of liso-cel for the treatment of adults with r/r CLL or small lymphocytic lymphoma. Similar to brexu-cel, non-T-cell elements are removed from the leukapheresis product prior to liso-cel manufacturing [9]. In TRANSCEND CLL 004, 117 patients, most of them resistant to both BTKi and venetoclax, were treated with liso-cel at two different DL. In the primary efficacy set at the optimum DL 2 (100 × 10^6^ CARTs; *n* = 49), ORR was 48% with a CR rate of 18%, resulting in a median PFS of 12 months. However, most of the complete responses were durable. In contrast, achieving a status of uMRD, which occurred in 76% of all patients evaluable for MRD, did not translate into superior PFS [52].

The low CR rate observed in these trials prompted us to attempt debulking of leukemia cell load prior to HD-CAR-1 treatment to facilitate CART efficacy and at the same time to reduce toxicity risks. In most heavily pretreated patients this was successfully achieved by using venetoclax-CD20 antibody combinations despite prior failure of fixed-duration venetoclax. Our data suggests that this can indeed result in high rates of CR or CR deepening with MRD clearance in the majority of patients treated at higher HD-CAR-1 dose levels. The CRs obtained or deepened with HD-CAR-1, however, mostly appeared to be less durable than those observed in the TRANSCEND CLL 004 trial. Application of HD-CAR-1 earlier during the treatment course and accompanying CLL-directed treatments, such as antibody or venetoclax maintenance and/or concomitant use of bispecific antibodies may be options for consolidating responses to HD-CAR-1 [53, 54].

Of note, no case of ICANS and only a single case of higher-grade CRS was observed with HD-CAR-1. This is in contrast to other CART trials in r/r CLL, where higher grade CRS and ICANS were observed much more frequently [51, 52, 55]. Although this might be partly related to the comparably low tumor burden of our patients at the time of CART infusion, it is in keeping with the favorable safety profile of HD-CAR-1 in ALL and lymphoma [29, 56] and points to the potential contribution of the third-generation design of HD-CAR-1 to its low toxicity. Despite extensive pretreatment with stem cell-toxic regimens such as fludarabine and cyclophosphamide (FC) and bendamustine and a HCT history in many patients, also ICAHT was extremely modest, again in line with previously published experience with HD-CAR-1 [29, 57].

CART products of HD-CAR-1 responders contained significantly more CD4 + T cells compared to non-responders. This observation is in keeping with Melenhorst et al. [58], who demonstrated that a CD4+ population dominated long-lasting CD19-directed CARTs in two CLL patients who showed a sustained CR for more than ten years. Furthermore, immunophenotyping of HD-CAR-1 CART products disclosed a strong enrichment of EM-like CD8 + T cells with high expression of CD39 and/or CD197 in non-responders compared to responders. This confirms the results of HD-CAR-1 in ALL patients, where a low CD39-expression on effector T cells within the CART product was associated with a higher response rate [29]. This clinical confirmation strengthens the role of CD39 within CART products as a marker for T-cell exhaustion [59, 60] and possible predictor of response in CART patients.

While the small sample size is an obvious limitation of this study, a major strength is its prospective design, demonstrating an extremely favorable safety profile and high response rate of the HD-CAR-1 approach tested here in heavily pretreated/double-refractory patients with CLL and, thus, potentially broadening patient eligibility for CART therapy in CLL.

In conclusion, treatment of heavily pretreated patients with high-risk CLL having failed multiple pathway inhibitors with the third-generation CART HD-CAR-1 is feasible and associated with only very modest CART-specific toxicity. The preliminary efficacy signals obtained suggest that HD-CAR-1 can induce prolonged complete responses in otherwise refractory patients and warrant further exploration of this approach.

## Supplementary information

SUPPLEMENTARY APPENDIX

## Data Availability

Original data are available with Patrick.Derigs@med.uni-heidelberg.de upon request.
