# Supplementary material for: Third-generation anti-CD19 CAR T cells for relapsed/refractory chronic lymphocytic leukemia: a phase 1/2 study
Source: Leukemia. 2024 Aug 27;38(11):2419–28. doi: 10.1038/s41375-024-02392-7 (PMC11519001; doi:10.1038/s41375-024-02392-7)
Supplement: Supplementary file 1 — SUPPLEMENTARY APPENDIX [file 41375_2024_2392_MOESM1_ESM.pdf]

SUPPLEMENTARY APPENDIX

Table of Contents:

Table S1. Staining antibodies used for analysis of cellular composition of CART products with flow cytometry.

Table S2. Characteristics and cellular composition of HD-CAR-1 CART products.

Table S1. Staining antibodies used for analysis of cellular composition of CART products with flow cytometry.

Antibodies	Vendor	Staining round
CD19-BUV496	BD	1
CAR T detection reagent	Miltenyi	2
CD16-BUV395	BD	3
CD33 BUV563	BD	3
CD314 BUV615	BD	3
CD27 BUV661	BD	3
CD8 BUV737	BD	3
CD45 BUV805	BD	3
CD141 BV421	Biolegend	3
IgD Pacific Blue	Biolegend	3
CD39 BV480	BD	3
ICOS BV510	BD	3
CD45RO BV570	Biolegend	3
CD11c BV605	Biolegend	3
CD279 BV650	BD	3
CD56 BV711	Biolegend	3
TCRab BV750	BD	3
CD45RA BV785	BD	3
CD11b BB515	BD	3
CD3 Spark Blue550	Biolegend	3
CD38 PerCP	Biolegend	3
CD94 BB700	BD	3
TCRgd PerCP-efluor710	Thermo Fisher	3
CD1c PE-Dazzle 594	Biolegend	3
CD95 PE-Fire640	Biolegend	3
ITGB7 PE-Cy5	BD	3
CD25 PE-Fire700	Biolegend	3
FcεR1α PE-Cy7	Invitrogen	3
CD4 PE-Fire810	Biolegend	3
CD197 APC	BD	3
CD123 Alexa Fluor 647	Biolegend	3
CD14 Spark-NIR 685	Biolegend	3
CD127 APC R700	BD	3
CD34 APC-Cy7	Biolegend	3
HLA-DR APC-Fire810	Biolegend	3
Zombie NIR (Fixable viability kit)	Biolegend	3
Brilliant Stain buffer plus	BD	3

Table S2. Characteristics and cellular composition of HD-CAR-1 CART products.

UPN	Total cells	Viability	$CD3^+$ CAR^+ T cells	$CD4^+$ T cells	$CD8^+$ T cells	$CD4^+$ $CD8^+$ T cells	$CD4^-$ $CD8^-$ T cells	$CD45RA^-$ $CD197^+$ T cells	$CD45RA^+$ $CD197^+$ T cells	$CD45RA^-$ $CD197^-$ T cells	$CD45RA^+$ $CD197^-$ T cells	Manufacturing (days)
1	298×10^6	94,9%	41,7%	63,3%	25,6%	0,8%	10,3%	23,6%	22,5%	33,1%	20,8%	10
2	107×10^6	86,2%	48,5%	39,8%	52,3%	5,2%	2,7%	27,8%	44,5%	12,7%	15,0%	10
3	599×10^6	91,7%	54,5%	60,4%	32,4%	1,9%	5,3%	34,4%	24,2%	25,5%	15,9%	10
4	1084×10^6	95,1%	67,7%	56,9%	39,8%	2,0%	1,4%	25,0%	19,7%	36,4%	18,9%	13
5	874×10^6	95,5%	62,3%	77,0%	16,7%	5,3%	1,0%	19,9%	16,2%	44,0%	19,9%	13
6	1432×10^6	95,6%	52,3%	50,7%	41,8%	1,7%	5,8%	17,3%	19,9%	38,6%	24,2%	13
7	840×10^6	95,8%	59,9%	62,3%	30,1%	0,5%	7,2%	19,7%	21,5%	39,6%	19,1%	13
8	922×10^6	95,5%	43,8%	71,7%	25,7%	0,5%	2,1%	19,8%	12,5%	54,5%	13,1%	13
9	1078×10^6	95,2%	50,5%	60,6%	37,0%	0,6%	1,8%	12,5%	5,5%	71,5%	10,5%	13

HD-CAR-1 CART products were analyzed with flow cytometry. Cells were stained with following antibodies: CD3 (clone SK7), CD4 (clone SK3), CD8 (clone SK1), CD45 (clone 2D1), CD45RA (clone HI100) and CD197 (clone G043H7). For detection of dead cells, LIVE/DEAD fixable NEAR-IR (Thermo Fisher Scientific), and for detection of CD19-directed HD-CAR-1 CAR positive cells, the biotinylated CD19 CAR Detection Reagent and the anti-biotin antibody (both Miltenyi Biotec) were applied. Samples were measured on BD FACSCanto Flow Cytometry Cell Analyzer (BD Biosciences). Data analysis was conducted with DIVA (BD Biosciences, version 8.0). Cells were counted with LunaFL Cell Counter (Logos Biosystems).
